# A functional type I interferon pathway drives resistance to cornea herpes simplex virus type 1 infection by recruitment of leukocytes^☆^

**DOI:** 10.1016/S1674-8301(11)60014-6

**Published:** 2011-03

**Authors:** Christopher D. Conrady, Heather Jones, Min Zheng, Daniel J.J. Carr

**Affiliations:** a Departments of Microbiology, Immunology, and; b Ophthalmology, The University of Oklahoma Health Science Center, Oklahoma City, OK 73104, USA

**Keywords:** herpes simplex virus type 1, type I interferon, cornea, viral infection, leukocytes, ocular immunology

## Abstract

Type I interferons are critical antiviral cytokines produced following herpes simplex virus type-1 (HSV-1) infection that act to inhibit viral spread. In the present study, we identify HSV-infected and adjacent uninfected corneal epithelial cells as the source of interferon-α. We also report mice deficient in the A1 chain of the type I IFN receptor (CD118^−/−^) are extremely sensitive to ocular infection with low doses (100 PFU) of HSV-1 as seen by significantly elevated viral titers in the cornea compared to wild type (WT) controls. The enhanced susceptibility correlated with a loss of CD4^+^ and CD8^+^ T cell recruitment and aberrant chemokine production in the cornea despite mounting an adaptive immune response in the draining mandibular lymph node of CD118^−/−^ mice. Taken together, these results highlight the importance of IFN production in both the innate immune response as well as eliciting chemokine production required to facilitate adaptive immune cell trafficking.

## INTRODUCTION

Herpes simplex virus type 1 (HSV-1) is a highly successful double-stranded DNA virus of which 60%-90% of the adult human population is seropositive[1]. During an acute infection, the lytic nature of the virus is driven by a sequential cascade of genes (referred to as lytic genes) expressed collectively over the course of the first 8-12 hrs following entry into the host cell and includes immediate early (α), early (β), and late (γ) genes[2]. It is now appreciated that many of these genes encode proteins that serve dual functions: assist in the replication of the virus and counter the innate or adaptive immune response to the virus. One such immediate early encoded protein, infected cell protein 0 (ICP0), is thought to act as a type I interferon (IFN) antagonist targeting Stat 1-driven, anti-viral pathway activation[3]. The central role of ICP0 as a viral-encoded immune countermeasure is underscored by the observation that in a fully immune competent host, ICP0 null (ICP0^−/−^) virus does not efficiently replicate whereas in a host lacking the functional type I IFN pathway, HSV-1 ICP0^−/−^ replication is not encumbered which ultimately leads to the demise of its host[3],[4]. Other HSV-1-encoded proteins ICP34.5 and Us11 antagonize a potent, IFN-inducible anti-viral pathway, double-stranded (ds) RNA activated protein kinase (PKR)[5], by direct interaction with dsRNA (in the case of Us11)[6], or uncoupling phosphorylation of the eukaryotic translation initiation factor 2 α[7]. Yet another HSV-1-encoded protein, ICP27, is found to interfere with NF-κB and IRF-3 signaling leading to a muted expression of IFN-β and other downstream IFN stimulatory genes[8]. As a result of a considerable effort on the part of the virus to block type I IFN expression and IFN-inducible pathways, it seems apparent that type I IFN expression is critical for the host to retain control of virus spread early during the initial rounds of replication and, thus, allow time for the development of an adaptive immune response to the pathogen.

Type I IFNs (IFN-α/β) not only activate a number of anti-viral pathways including RNase L, PKR, and Mx protein GTPases[9]–[11], but they also augment maturation of dendritic cells and activate natural killer (NK) cells, dendritic cells, B and T lymphocytes, and maintain and facilitate clonal expansion of activated T cells[12]–[15]. Moreover, expression of type I IFN contributes to the development of memory CD4^+^ and CD8^+^ T cells; two cellular components that are likely critical in the generation of a successful vaccine targeting viral pathogens[16],[17]. Previously, we reported mice deficient in the type I IFN receptor alpha1 chain (CD118^−/−^) were highly susceptible to ocular HSV-1 infection[18]. The heightened sensitivity was associated with a total collapse in the T cell population residing in the draining lymph nodes as well as a loss of T cell migration into the nervous system[18]. This loss in T cell number was due, in part, to early apoptosis of draining lymph node T cells that also corresponded to dissemination of the virus. CD118^−/−^ mice succumbed to infection by day 5-6 post virus exposure which precluded the ability to fully evaluate the development of antigen-specific CD8^+^ T cells in organized lymphoid tissue or at inflamed sites. To prolong survival of the CD118^−/−^ mice to infection and evaluate the adaptive immune response, the infectious dose was reduced 10-fold from that previously published[18]. It was hypothesized that by prolonging survival the adaptive immune response would have time to develop, which would allow us to more fully characterize the host response. We found that even though the adaptive T cell response was similar in the draining lymph node comparing wild type to CD118^−/−^ mice, the CD118^−/−^ animals were still highly susceptible to infection as a result of the inability of T cells to traffic to local sites of infection.

## MATERIALS AND METHODS

### Mice and virus

C57BL/6J (WT) mice were purchased from The Jackson Laboratory. Mice deficient in the type I IFN receptor alpha chain (CD118^−/−^)[19] on a WT background were maintained in the OUHSC barrier facility. Animal treatment was consistent with the National Institutes of Health Guidelines on the Care and Use of Laboratory Animals. All procedures were approved by the University of Oklahoma Health Sciences Center and Dean A. McGee Eye Institute Institutional Animal and Care Use Committees. HSV-1 (strain McKrae) was grown and maintained as previously described[20].

### HSV-1 infection

Male WT and CD118^−/−^ mice (6-10 wks of age) were anesthetized by i.p. injection with xylazine (6.6 mg/kg) and ketamine (100 mg/kg) followed by scarification of the cornea using a 27 gauge needle. The tear film was removed using tissue (Kimwipe), and the cornea was topically inoculated with 100 plaque forming units (PFU) of HSV-1 in 3.0 µL of RMPI-1640 medium. Viral titers were determined in the cornea, trigeminal ganglia (TG), and brain stem (BS) at d 3 or d 6 post infection (PI) by plaque assay as previously described[21].

### Flow cytometry

Mice were exsanguinated at day 7 PI and the cornea and mandibular lymph nodes (MLN) were removed, processed, labeled with antibody or HSV glycoprotein B (gB) tetramer, and analyzed using a Coulter Epics XL flow cytometer (Beckman, USA), and the absolute number of cells residing in the tissue or lymph node was determined as previously described[21].

### ELISA

At d 7 PI, the corneas of exsanguinated infected mice were removed, weighed, and placed in 500 µL of 1× PBS containing protease cocktail inhibitor mixture (Calbiochem, USA) on ice. Following homogenization with a tissue miser (Fisher Scientific, USA), the homogenates were clarified by centrifugation (10,000 *g*, 1 min). The levels of CXCL1 and CXCL10 were determined by ELISA according to the manufacturer's instructions (Quantikineimmunoassay; R&D Systems, USA).

### Confocal microscopy

WT mice were infected with 1,000 PFU/cornea. Three days PI, mice were euthanized and their corneas harvested. The corneas were fixed in 4% paraformaldehyde for 30 min at room temperature. Following three washes of 30 min each with 1× PBS-Triton-X100, corneas were suspended in 100 mL of 1× PBS-BGEN containing 1 mL of donkey serum (Jackson Immuno Labs, USA) to minimize nonspecific antibody binding and incubated at 4°C overnight (O/N). Serum was then removed and corneas were subjected to polyclonal rabbit anti-HSV-1 (Dako Cytomation, USA) in 1× PBS-BGEN at 4°C O/N. Corneas were then washed 3 times for 30 min each with 1× PBS-Triton-X100 and then stained with a secondary labeled DyLight 549 anti-rabbit (Jackson Immuno Labs, USA) and a FITC-conjugated rat anti-mouse monoclonal IgG to either IFN-α, -β, or isotype control (PBL Biomedical Laboratories, USA). After O/N incubation at 4°C, corneas were washed again as previously described and submerged in DAPI (Vector, USA) for 24 hours. Stained corneas were then mounted and imaged with an Olympus IX81-FV500 epifluorescent/confocal laser-scanningmicroscope at 200× magnification with a digital zoom of 3×. Captured images were then analyzed with Fluoview software (Olympus, USA). Three dimensional reconstructions of the cornea were performed using a step size of 0.6 mm in the z-axis and then combined with Fluoview software.

### Statistics

Statistical analysis was conducted using the GB-STAT program (Dynamic Microsystems, USA). Student's *t*-test was used to determine significant (*P* < 0.05) differences between WT and CD118^−/−^ mice.

## RESULTS

### Interferon-α is expressed in HSV-1-infected and adjacent uninfected epithelial cells

**Fig. 1:**
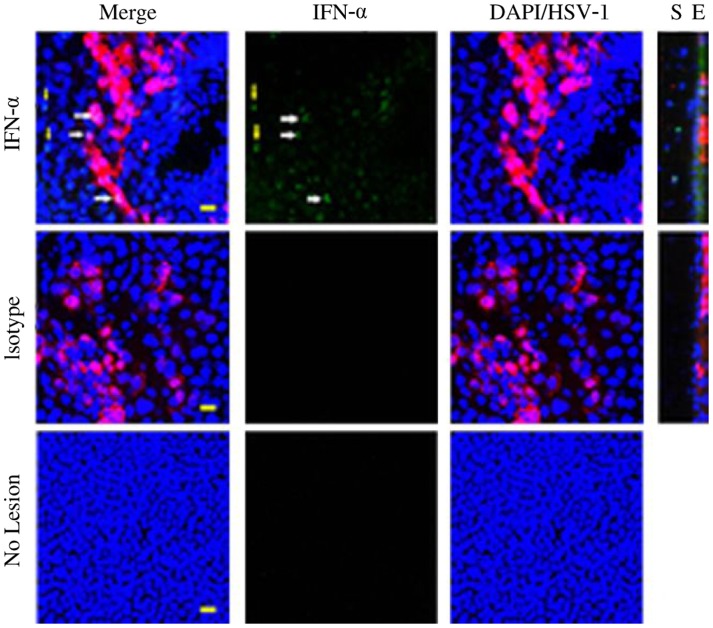
Expression of IFN-α by corneal epithelial cells following HSV-1 infection. Anesthetized wild type WT mice (*n* = 8 / stain) were infected with 1,000 PFU / cornea HSV-1. At d 3 PI, corneas were harvested, fixed, and stained for HSV-1 (red), IFN-α (green), and cell nuclei (blue). White arrows highlight infected cells expressing IFN-α, while yellow arrows indicate non-infected, IFN-α producing cells. The top panel indicates IFN-α staining at sites of infection, the middle images are of IFN-α isotype controls, and the bottom pane is IFN-α staining at uninfected sites. On the far right are 3D reconstructions of the corneas on the left in which the top picture indicates IFN-α staining (in green), while the lower image is the isotype control. Images are representative of four independent experiments (2 corneas/group/experiment). Red represents HSV-1 antigen expression. S: corneal stroma; E: corneal epithelium; Yellow bar=50 µm.

The use of neutralizing antibody to IFN-α/β has previously been used to demonstrate a role for type I IFN in resistance to HSV-1 infection following ocular inoculation[22],[23]. Additional studies have found RNase L, a downstream effector pathway elicited by type I IFNs, is activated in the cornea following HSV-1 infection[24]. However, no studies have formally demonstrated the location of type I IFN expression within the cornea following HSV-1 infection. In order to define the site of type I IFN production relative to HSV-1, imaging analysis was conducted on the cornea following virus infection. IFN-α expression was consistently found within the epithelial layer of the cornea following acute infection (***Fig. 1***). The expression of the cytokine co-localized with HSV-1 antigen expression with some non-infected cells adjacent to the lesion also expressing IFN-α. In contrast to IFN-α, IFN-β was not consistently detected in the cornea proper at d 3 PI (data not shown). However, IFN-β was detectable within the limbus (peripheral of the cornea proper) of the anterior segment of the eye. Therefore, the notion that type I IFN may influence the outcome of a viral corneal infection is supported by expression of the cytokine within the lesion generated from replicating virus.

### HSV-1 spreads rapidly to the nervous system in the absence of a functional type I IFN-pathway

**Fig. 2:**
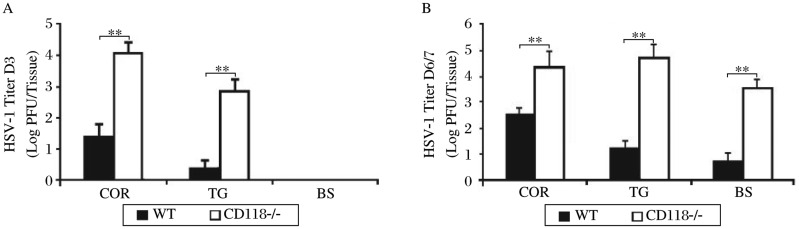
HSV-1 replication is augmented in the absence of a functional type I IFN pathway. Anesthetized wild type (WT) and CD118^−/−^ mice (*n* = 6/group) were infected with 100 PFU/cornea HSV-1. At d 3 (A) or d 6-7 (B) PI, the mice were exsanguinated and the cornea (COR), trigeminal ganglion (TG), and brain stem (BS) were removed, processed, and assayed for viral content by plaque assay. The bars represent the mean±SE summarizing two experiments. **P < 0.01 comparing the WT to CD118^−/−^ groups for each corresponding tissue.

Following local replication within the cornea, HSV-1 spreads to the nervous system by retrograde transport initially colonizing the trigeminal ganglion (TG)[25]. In a previous study, it was reported virus trafficked to the TG of WT and CD118^−/−^ mice at d 3 PI with more virus recovered from the tissue of CD118^−/−^ mice in comparison to WT animals following the application of an infectious inoculum of 1,000 PFU/cornea. In the present study, even at a lower infectious dose of 100 PFU/cornea, CD118^−/−^ mice were highly sensitive to HSV-1 infection. Specifically, at d 3 PI, 100% of tissue samples obtained from the cornea and TG of CD118^−/−^ mice harbored infectious virus compared to 100% of cornea samples and 33% of TG samples obtained from WT mice. Moreover, there was a significant increase in the viral titer found in each tissue from CD118-/- mice (***Fig. 2A***). Similar results were found at d 6 PI as well (***Fig. 2B***). The data underscore the contribution type I IFN has as an effective component of the host's immune repertoire in controlling local HSV-1 replication and spread during acute infection of the eye.

### Deficiency of T cell recruitment to the cornea of HSV-1-infected CD118^−/−^ mice

**Fig. 3:**
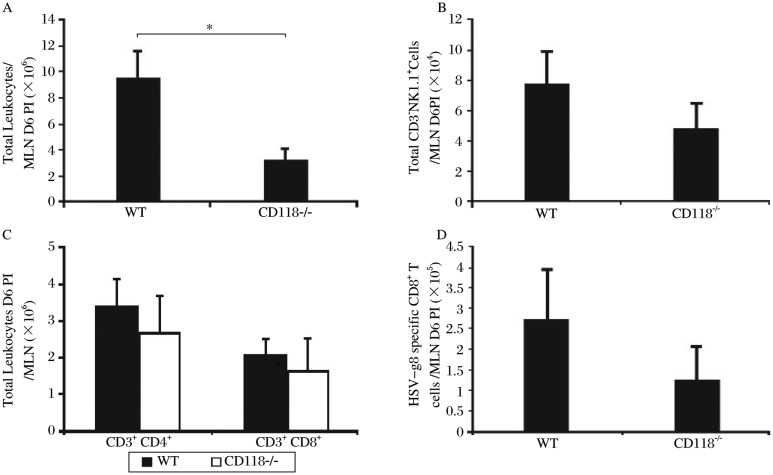
Loss of total leukocyte population in the draining lymph node of HSV-1-infected CD118^−/−^ mice. Anesthetized wild type (WT) and CD118^−/−^ mice (*n* = 6/group) were infected with 100 PFU/cornea HSV-1. At d 6 PI, the mice were exsanguinated and the mandibular lymph nodes (MLN) were removed, processed, and analyzed for total (CD45^hi^) leukocyte (A), NK cell (B), T cells (C), and HSV gB-specific CD8^+^ T cells (D) by flow cytometry. The bars represent the mean±SEM summarizing two experiments. *P < 0.05 comparing the WT to CD118^−/−^ total leukocyte population.

The expression of type I IFNs is known to influence the transition of the cell-mediated immune response through activation and mobilization of NK cells, distribution of lymphocytes, and acquisition of T cell effector function[26]–[29]. In a previous study using CD118^−/−^ mice, a viral inoculum of 1,000 PFU/cornea resulted in the death of mice prior to the development of an adaptive immune response[18]. Given sufficient time to develop an adaptive immune response, the question was asked as to whether in the absence of a functional type I IFN pathway an antigen-specific T cell response could develop and contribute toward resistance to the pathogen. Initially, we investigated the phenotypic profile of immune cells residing in the draining lymph node (mandibular lymph node, MLN) of CD118^−/−^ mice compared to the WT counterparts d 6-7 PI. The total leukocyte population (CD45^hi^) was reduced 2.5 fold in the CD118^−/−^ MLN (***Fig. 3A***). However, there were no significant differences in the absolute number of NK cells (***Fig. 3B***), total CD4^+^ or CD8^+^ T cells (***Fig. 3C***), or HSV-specific CD8^+^ T cells in the MLN of CD118^−/−^ mice compared to the WT animals. Therefore, the absence of a functional type I IFN pathway did not negatively influence the development of the adaptive immune response within the organized lymphoid tissue of the CD118^−/−^ mice at a lower infectious inoculum of 100 PFU/cornea compared to the previous 1,000 PFU/cornea dose[25].

**Fig. 4:**
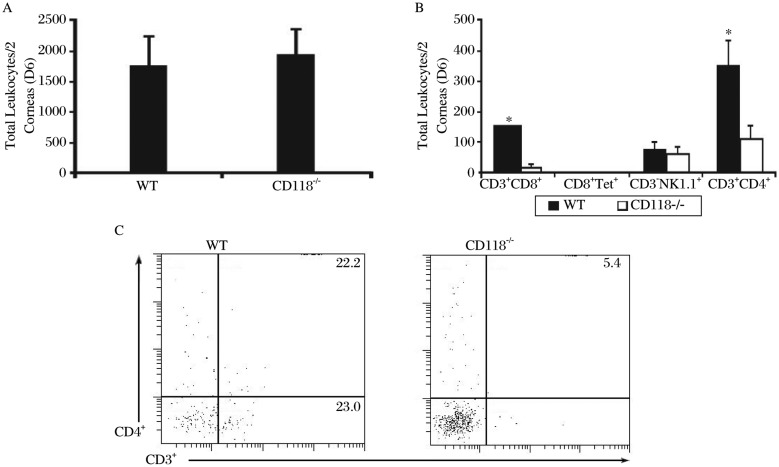
Deficiency of T cell recruitment to the cornea of HSV-1-infected CD118^−/−^ mice. Anesthetized wild type (WT) and CD118^−/−^ mice (*n* = 6/group) were infected with 100 PFU/cornea HSV-1. At d 6 PI, the mice were exsanguinated and the corneas were removed, processed, and analyzed for total (CD45hi) leukocyte (A) and NK cell, T cell, and HSV gB-specific CD8^+^ T cells (B) by flow cytometry. C: A representative flow cytometry result is included depicting a representative analysis of CD3^+^CD4^+^ T cells from WT and CD118^−/−^ mice. The bars represent the mean±SE summarizing two experiments. *P < 0.05 comparing the WT to CD118^−/−^ groups for CD3^+^CD4^+^ and CD3^+^CD8^+^ T cell populations.

Since the development of the adaptive immune response was spared in the MLN of CD118^−/−^ mice following HSV-1 infection, the infiltration of the corresponding cells within the cornea was next investigated. Whereas there was no difference in the total number of leukocytes (***Fig. 4A***) or NK cells (***Fig. 4B***) populating the cornea of WT versus CD118^−/−^ mice, total CD4^+^ and CD8^+^ T cells were grossly deficient or nearly absent in the cornea of CD118^−/−^ mice (***Fig. 4B***). There were no detectable HSV-specific CD8^+^ T cells either (***Fig. 4B***). Consequently, the lack or reduction of T cells populating the cornea in response to HSV-1 may contribute to the increased sensitivity to the virus in the CD118^−/−^ mice.

**Fig. 5:**
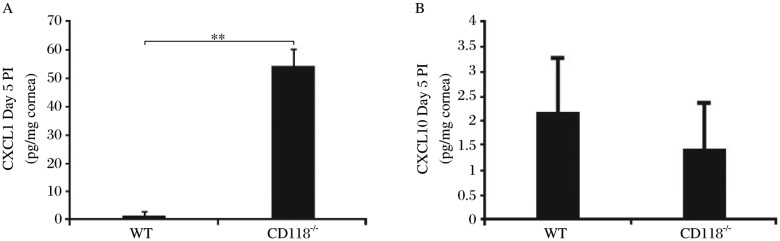
CXCL1 and CXCL10 levels are modified in the cornea of CD118-/- mice in response to HSV-1 infection. Anesthetized wild type (WT) and CD118^−/−^ mice (*n* = 9/group) were infected with 100 PFU/cornea HSV-1. At d 5 PI, the mice were exsanguinated and the corneas were removed, processed and analyzed for CXCL1 (A) and CXCL10 (B) protein content by ELISA. The bars represent the mean±SEM summarizing three independent experiments. **P < 0.01 comparing WT and CD118^−/−^ mice.

The principal soluble mediators in the recruitment of leukocytes to the cornea of HSV-1-infected mice are chemokines[30],[31]. One of the primary chemokines expressed early in the cornea in response to HSV-1 infection is CXCL10[32]. Antibody neutralization targeting CXCL10 was found to significantly reduce leukocyte infiltration into the cornea in response to HSV-1[33]. Therefore, we investigated the expression of CXCL10 in the cornea of WT and CD118^−/−^ mice following HSV-1 infection. In contrast to our hypothesis, CXCL10 expression was not reduced in the CD118^−/−^ mice in comparison to WT controls (***Fig. 5A***). In contrast, another chemokine, CXCL1, was elevated in the cornea of CD118^−/−^ mice (***Fig. 5B***). Taken together, the results depict a selective mechanism of induction of chemokine expression based on the presence of a functional type I IFN pathway.

## DISCUSSION

Type I IFN receptor null mutant mice (CD118^−/−^) show abnormalities in responses of hematopoietic cells as well as high sensitivity to viral pathogens[34],[35]. In the absence of a functional type I IFN receptor, HSV-1 infection of mice results in rapid dissemination of the virus throughout the animal including liver involvement[36]. This outcome is not surprising as type I IFNs are activated by innate sensors in driving host anti-viral defense acutely and greatly contribute to the development of T cell-dependent antibody responses that are also known to influence viral clearance[37],[38]. Within the cornea, ectopic expression of IFN-α or IFN-β markedly suppresses HSV-1 replication and spread associated with a reduction in lytic gene expression[39],[40]. Although indirect evidence suggests type I IFNs are active within the cornea[3], up to this point there are no studies that have described the location of IFN-α or IFN-β expression in response to HSV-1 infection. To the best of our knowledge this is the first report that defines the expression of IFN-α localized to the corneal epithelial cells principally infected with the virus. Surprisingly, we did not consistently find IFN-β expression except peripheral to the cornea proper in the limbal zone. The cornea is comprised of three principal layers including the stratified non-keratinized squamous epithelium, a multi-layered stroma, and a single endothelial layer that lines the anterior chamber of the eye[41]. Immature myeloid-derived dendritic cells have been reported to reside in the stromal layer[42]. Likewise, macrophage-type cells (defined as CD11b^+^CD11c^–^) have been identified in the posterior stroma of the non-inflamed, normal cornea[43]. Within the corneal epithelium, Langerhans cells have been identified[44] that in fact, could respond to HSV-1 infection with the expression of type I IFN. In the current study, the analysis of IFN-α expression in the epithelium following HSV-1 infection cannot rule out involvement of the Langerhans cells in type I IFN production as they may be masked by epithelial cell expression of the cytokine. However, we can confidently conclude that corneal epithelium is one source of IFN-α during the initial acute virus infection without a contribution by IFN-β.

In the present study, total CD4^+^ and CD8^+^ T cell infiltration into the cornea of CD118^−/−^ mice in response to HSV-1 was compromised compared to that found in the cornea of WT mice. This difference was not reflected by the development of the adaptive immune response in terms of T cells populating the draining (mandibular) lymph node following infection. Specifically, in the absence of infection, CD4^+^ and CD8^+^ T cells maintain close to one million cells/MLN in WT and CD118^−/−^[18] whereas following 100 PFU/cornea HSV-1, similar levels (2-3 million) of CD4^+^ and CD8^+^ T cells were isolated from the MLN of WT and CD118^−/−^ mice 6 d PI. We interpret these results to indicate the response to antigen/virus within the draining lymph nodes is similar between WT and CD118^−/−^ mice. Consequently, the T cell deficiency found in the cornea of CD118^−/−^ animals must be due to an inability to attract the cells to the site of inflammation. To address this possibility, the expression of one of the primary chemokines associated with T cell recruitment, CXCL10[45], was investigated. CXCL10 levels were not found to be lower in CD118^−/−^ mice in response to viral challenge dissimilar to what we previously reported using a higher viral dose[46]. In addition to CXCL10, other chemokines including CXCL9 which is also diminished in the absence of a functional type I IFN pathway[46] and is thought to promote CD4^+^ T cell recruitment to the cornea following HSV-1 infection[47] may be driving T cell recruitment. In contrast to CXCL10, CXCL1 levels were elevated in CD118^−/−^ mice. CXCL1 has previously been associated with HSV-1 infection as it is known to chemoattract neutrophils[30]. In the nervous system, CXCL1 is strongly expressed in HSV-1-infected CD118^−/−^ mice commensurate with viral load in comparison to WT animals[18]. We would submit that the expression of this chemokine reflects the antigen load which can activate innate immune sensors (e.g., TLR3, TLR7, or TLR9) and drive NFκB activation which then induces CXCL1 expression[48]. The impact of heightened CXCL1 levels in the cornea of CD118^−/−^ mice has not yet been investigated.

T cells are a critical component in the control of HSV-1 infection[49],[50]. Within the cornea CD4^+^ and CD8^+^ T cells dampen HSV-1 replication through the production of cytokines including IFN-γ or direct cytolysis of virally infected cells that limit the production of infectious virions[51]–[54]. In the present study, both CD4^+^ and CD8^+^ T cell numbers were reduced in the cornea of CD118^−/−^ mice by d 6-7 PI. However, there were modest levels (i.e., < 20) of HSV gB-specific CD8^+^ T cells residing in the cornea of WT mice at this time suggesting their contribution in the control of HSV-1 was likely minimal. By increasing the viral inoculum to 1,000 PFU/cornea, HSV gB-specific CD8^+^ T cells are readily detected in the cornea by day 7 PI in WT mice and do contribute to viral surveillance (Conrady and Carr, manuscript in preparation). Since there was no difference in NK cell numbers comparing the WT to CD118^−/−^ mice, one assumption is that this population is not a significant contributing party in resistance to virus replication in the cornea during acute infection. However, NK cells contribute to resistance to HSV-1 infection in the eye in the absence of T cells and are responsive to type I IFN in terms of activation. Therefore, it is likely that even though similar levels of NK cells exist in the cornea of CD118^−/−^ mice relative to WT animals, the activation status of these cells as it relates to effector function (including IFN-γ production and cytolytic activity) is muted.

In summary, the current study has demonstrated susceptibility to a low level virus infection of the cornea cannot be overcome in the absence of a functional type I IFN pathway. Not only does the virus replicate locally but disseminates rapidly to the nervous system in the absence of the type I IFN receptor alpha 1 chain. Phenotypically, the development of the adaptive immune system is similar in the MLN of WT and CD118^−/−^ mice at this level of infection but the T cells are not recruited in sufficient numbers to the cornea to assist the resident and innate immune cell resistance to the pathogen. We conclude that the fate of resistance in terms of type I IFN expression within the cornea operates at two levels. The first is direct opposition to lytic gene expression through the activation of intracellular effector mechanisms including the activation of PKR and RNase L pathways that target the temporal expression of viral genes and protein at the transcriptional or translational level. The second form of resistance is indirect via the induction of chemokines that assist in the recruitment of effector T cells and likely facilitates the activation of NK cells. This combinatorial process significantly reduces the replication and spread of the virus from the cornea to the nervous system where the virus can establish a latent infection in the neurons within the sensory ganglion or further travel to the brain ultimately resulting in encephalitis and death of the animal. Whether or not type I IFNs are necessary to drive HSV-1 from a lytic to a latent infection is currently unknown since CD118^−/−^ mice succumb to infection even following the administration of neutralizing HSV-1 antibody or acyclovir treatment that stops HSV-1 replication in WT mice. It would be of interest to determine if HSV-1 can be driven into latency by other means in CD118^−/−^ mice in order to evaluate the degree of latent infection in the neurons as well as the incidence of spontaneous versus induced reactivation.
